# Effects of exogenous oxytocin and estradiol on resting-state functional connectivity in women and men

**DOI:** 10.1038/s41598-023-29754-y

**Published:** 2023-02-22

**Authors:** Marie Coenjaerts, Berina Adrovic, Isabelle Trimborn, Alexandra Philipsen, René Hurlemann, Dirk Scheele

**Affiliations:** 1 Research Section Medical Psychology, Department of Psychiatry and Psychotherapy, University Hospital Bonn, 53105 Bonn, Germany; 2 Department of Psychiatry and Psychotherapy, University Hospital Bonn, 53105 Bonn, Germany; 3 Department of Psychiatry, School of Medicine & Health Sciences, University of Oldenburg, 26129 Oldenburg, Germany; 4 Research Center Neurosensory Science, University of Oldenburg, 26129 Oldenburg, Germany; 5 Department of Social Neuroscience, Faculty of Psychology, Ruhr-University Bochum, 44780 Bochum, Germany

**Keywords:** Neuroscience, Endocrinology, Brain

## Abstract

Possible interactions of the neuropeptide oxytocin and the sex hormone estradiol may contribute to previously observed sex-specific effects of oxytocin on resting-state functional connectivity (rsFC) of the amygdala and hippocampus. Therefore, we used a placebo-controlled, randomized, parallel-group functional magnetic resonance imaging study design and measured amygdala and hippocampus rsFC in healthy men (n = 116) and free-cycling women (n = 111), who received estradiol gel (2 mg) or placebo before the intranasal administration of oxytocin (24 IU) or placebo. Our results reveal significant interaction effects of sex and treatments on rsFC of the amygdala and hippocampus in a seed-to-voxel analysis. In men, both oxytocin and estradiol significantly decreased rsFC between the left amygdala and the right and left lingual gyrus, the right calcarine fissure, and the right superior parietal gyrus compared to placebo, while the combined treatment produced a significant increase in rsFC. In women, the single treatments significantly increased the rsFC between the right hippocampus and the left anterior cingulate gyrus, whereas the combined treatment had the opposite effect. Collectively, our study indicates that exogenous oxytocin and estradiol have different region-specific effects on rsFC in women and men and that the combined treatment may produce antagonistic effects.

## Introduction

The hypothalamic peptide oxytocin (OXT) has a broad profile of effects ranging from labor induction and lactation^1^ to social approach and avoidance behavior^2–4^, romantic attachment^5–9^, as well as fear^10–13^ and trauma processing^14,15^. Neural effects of OXT in the hippocampus and amygdala are in line with higher expression of OXT receptors in these subcortical areas^1,16^. Furthermore, intranasal OXT has been found to modulate the functional communication between and within large-scale brain networks during resting state measured with electroencephalography^17^ and functional magnetic resonance imaging (fMRI)^18–21^. Importantly, there is accumulating evidence that OXT exhibits sex-specific effects on neural responses during the perception or evaluation of socio-emotional stimuli^20,22–27^ and on resting-state fMRI^28–30^. Preliminary evidence indicates that OXT affects resting-state functional connectivity (rsFC) between and within emotion and reward-related networks including the amygdala in a sex-dependent manner^28,31^. Additionally, as the amygdala is a set of functionally heterogeneous nuclei, a subregional-specific modulatory role of OXT on amygdala-centered emotion processing networks has been suggested^32,33^, but sex-specific effects on rsFC of amygdala subregions have not been examined yet. Potential mechanisms contributing to the sex differences in rsFC include menstrual cycle effects and the interaction of OXT with sex hormones such as estradiol (EST)^28–30,34^.

Fluctuations of sex hormones along the menstrual cycle impact rsFC^35^ and effects of EST appear to be pronounced for the amygdala and the hippocampus. Both regions express a high density of EST receptors^36^ and are sensitive to changes in estrogens^37,38^. For instance, elevated EST levels are related to an increase in hippocampal and amygdala gray matter volume^39–42^. In addition, higher EST levels positively correlate with hippocampal^39,43,44^ and amygdala rsFC^45^. As yet, no study has probed the effects of exogenous EST on rsFC in men, but a recent study found that a single dose of exogenous testosterone modified the rsFC of the amygdala^46^. Given that testosterone is catalyzed to estrogen via the enzyme aromatase^47^, an effect of exogenous EST on rsFC in men is therefore conceivable.

Evidence for EST-OXT interactions derive from animal models showing that EST and OXT modulate the synaptic plasticity in the medial nucleus of the amygdala in male rats^48^ and that EST receptors induce an OXT production by binding in a dimerized form to the composite hormone response element of the OXT promotor gene^49–51^. Importantly, also in humans possible EST-OXT interactions have been discussed for various domains including social anxiety and migraine attacks^2,52^. There is preliminary evidence deriving from task-based fMRI studies that EST and OXT may antagonistically interact^23,53^. However, no study has simultaneously probed the modulatory effects of exogenous EST and OXT on rsFC and possible interactions in women and men. Over recent decades, large resting state fMRI (rsfMRI) datasets have been collected in neuroimaging consortia (e.g. the UK Biobank^54^) to decipher the functional integration of brain regions into interconnected networks. Machine learning approaches have been applied to rsfMRI data to predict demographic characteristics such as sex^55^ and age^56^. Importantly, rsfMRI can help to identify neurophysiological subtypes of neuropsychiatric disorders like depression and these biomarkers may be useful to predict treatment responses^57^. For possible future clinical applications, a better understanding of sex differences and hormonal interaction effects is crucial. Previous studies observed that changes of rsFC significantly correlate with hormonal changes across the menstrual cycle^36^, but the simultaneous alterations of multiple hormones hamper causal attributions. Furthermore, these findings cannot be extrapolated to men. By exploring the effects of exogenous OXT and EST on rsfMRI in women and men, we can overcome these limitations and control for possible context-dependent effects of OXT which have become evident in task-based studies. For instance, it has been found that OXT effects on amygdala activation are dependent on the emotional valence of faces^58–60^ (but see also^61^), while other neural effects were moderated by previous experiences with the shown social stimuli^6,8^. Therefore, we conducted a pre-registered, randomized, placebo-controlled, parallel-group fMRI study involving healthy men and free-cycling healthy women in their early follicular phase to test the effects of exogenous EST, OXT, and their interaction on rsFC. Participants were scanned under four experimental conditions: 1. Transdermal placebo gel and intranasal placebo (PLC_tra_ & PLC_int_), 2. Transdermal placebo and intranasal OXT (PLC_tra_ & OXT_int_), 3. Transdermal EST and intranasal placebo (EST_tra_ & PLC_int_), and 4. Transdermal EST and intranasal OXT (EST_tra_ & OXT_int_).

Our first hypothesis was that under placebo men and women would differ in their amygdala and hippocampus rsFC^62–65^. We further expected that OXT_int_ would have opposing effects on hippocampal and amygdala rsFC in women and men^23,29,31^. However, due to the mixed results of OXT_int_ effects (e.g., increased and decreased rsFC), we refrained from formulating a directed hypothesis. Additionally, the observed correlation between higher EST levels and increased amygdalar and hippocampal rsFC^40,44–46^, led to the hypothesis that the EST_tra_ treatment would increase the amygdalar and hippocampal rsFC in women and alter it in men. Due to limited research on EST effects in men, we abstained from hypothesizing a direction for the EST_tra_ effects in men. Furthermore, due to the possible antagonistic relation between OXT and EST, we hypothesized that the single treatment effects of either EST_tra_ or OXT_int_ would be reduced or inverted in the combined treatment group in both sexes^23^. In additional explorative analyses, we examined possible treatment and sex effects on the rsFC of amygdala subregions^33,34^. Furthermore, given that both OXT^66,67^ and EST^68^ have been found to modulate the default mode network (DMN), we also explored effects on rsFC of the DMN.

## Results

### Sex differences under placebo

To probe sex differences under placebo, we examined rsFC of the hippocampus, the amygdala, and the amygdala subregions as separate seed regions in a seed-to-voxel analysis (cluster defining threshold *p* < 0.001; significance threshold *p* < 0.05 false discovery rate-corrected, *p*_FDR_) in placebo-treated (PLC_tra_ & PLC_int_) women and men. Analyses revealed that women showed a decreased rsFC between the right hippocampus and the left anterior cingulate gyrus in contrast to men (MNI_xyz_: - 4, 40, 26, *k* = 133, *p*_FDR_ = 0.002). The rsFC of the left hippocampus and the bilateral amygdala were not significantly different between the sexes under PLC (all *ps* > 0.05). However, an analysis of the amygdala subregions showed that for the right superficial amygdala as a seed region, the rsFC with the left cerebrum was increased in men compared to the women (MNI_xyz_: - 32, 10, 18, *k* = 80, *p*_FDR_ = 0.03).

### Significant sex * EST_tra_ treatment * OXT_int_ treatment interactions

We observed significant sex * EST_tra_ treatment * OXT_int_ treatment interactions in rsFC of both the hippocampus and the amygdala as separate seed regions in a seed-to-voxel analysis. Significant interactions were identified in rsFC of the right hippocampus with the left anterior cingulate gyrus (MNI_xyz_: - 4, 38, 26, *k* = 98, *p*_FDR_ = 0.02) and in rsFC of the left amygdala with the right lingual gyrus (MNI_xyz_: 16, - 52, 00, *k* = 147, *p*_FDR_ = 0.002) and the left cuneus (MNI_xyz_: - 16, - 78, 16, *k* = 88, *p*_FDR_ = 0.02). There was no significant three-way interaction for the right amygdala or the left hippocampus. Additional analyses of the amygdala subregions revealed significant three-way interactions in rsFC of the left centromedial amygdala with the left cuneus (MNI_xyz_: - 16, - 78, 16, *k* = 122, *p*_FDR_ = 0.003), the left lingual gyrus (MNI_xyz_: - 20, - 50, - 4, *k* = 112, *p*_FDR_ = 0.003), and the right calcarine gyrus (MNI_xyz_: 24, - 50, 4, *k* = 173, *p*_FDR_ < 0.001). By contrast, for the right superficial amygdala as a seed region, we observed a significant three-way interaction on rsFC with the right frontal lobe (MNI_xyz_: 20, 36, 00, *k* = 89, *p*_FDR_ = 0.047). Importantly, we examined whether these group effects might be driven by motion and we observed no significant main or interaction effects with respect to the mean framewise-displacement (all *p*s > 0.05). The reported significant interaction effects are decomposed by examining treatment effects within the sexes.

### Treatment effects within the sexes

**Figure 1:**
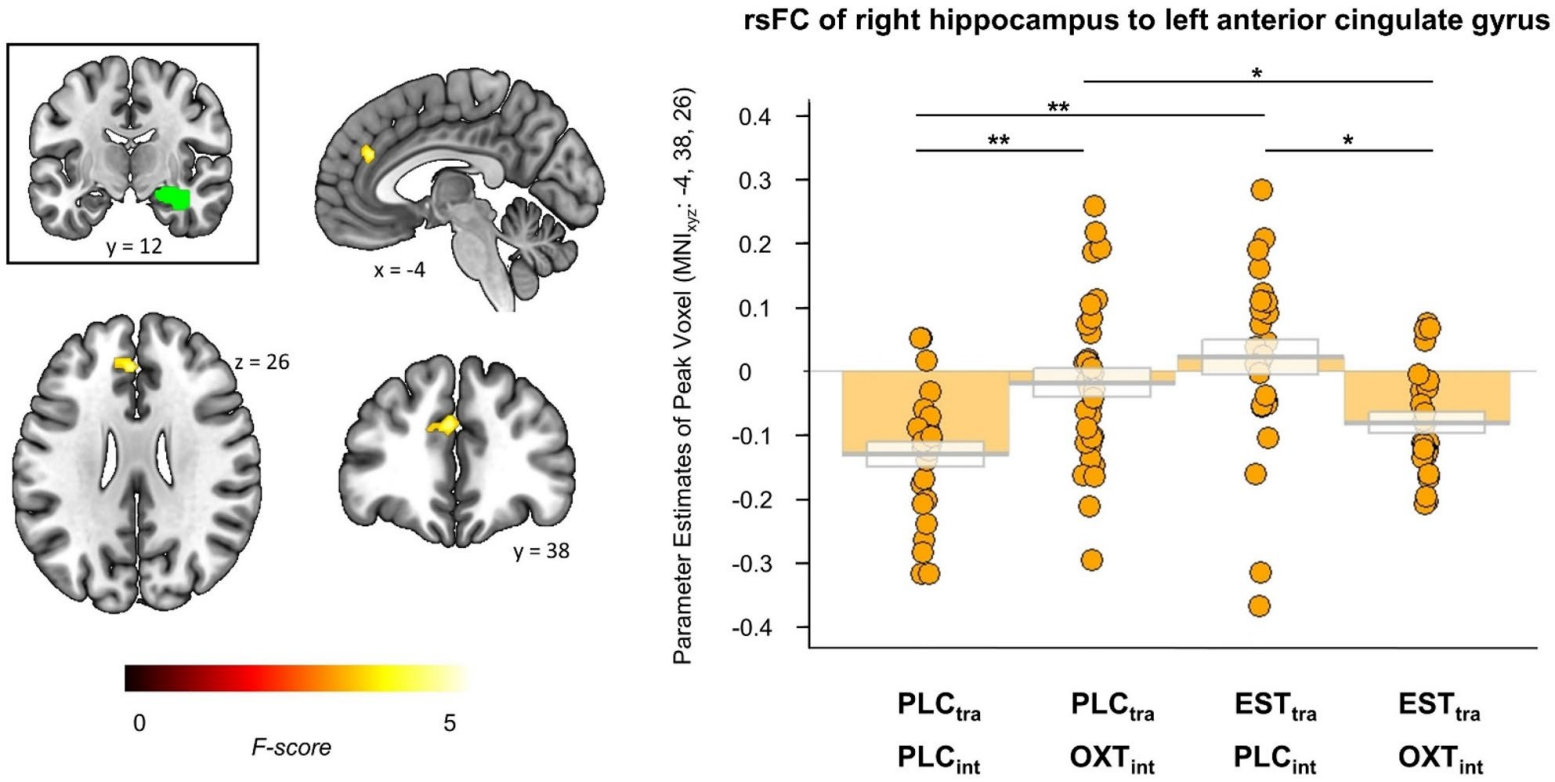
Treatment effects on the resting-state functional connectivity (rsFC) between the right hippocampus (green cluster) and the left anterior cingulate gyrus in women. The single treatment with either estradiol or oxytocin significantly increased rsFC between the right hippocampus and the left anterior cingulate gyrus. However, the combined treatment led to an rsFC between the right hippocampus and the left anterior cingulate gyrus comparable to that of the placebo group. Error bars indicate standard errors of the mean. PLC_tra_ = transdermal placebo gel; PLC_int_ = intranasal placebo; OXT_int_ = intranasal oxytocin; EST_tra_ = transdermal estradiol. *p < 0.05, **p < 0.01.

**Figure 2:**
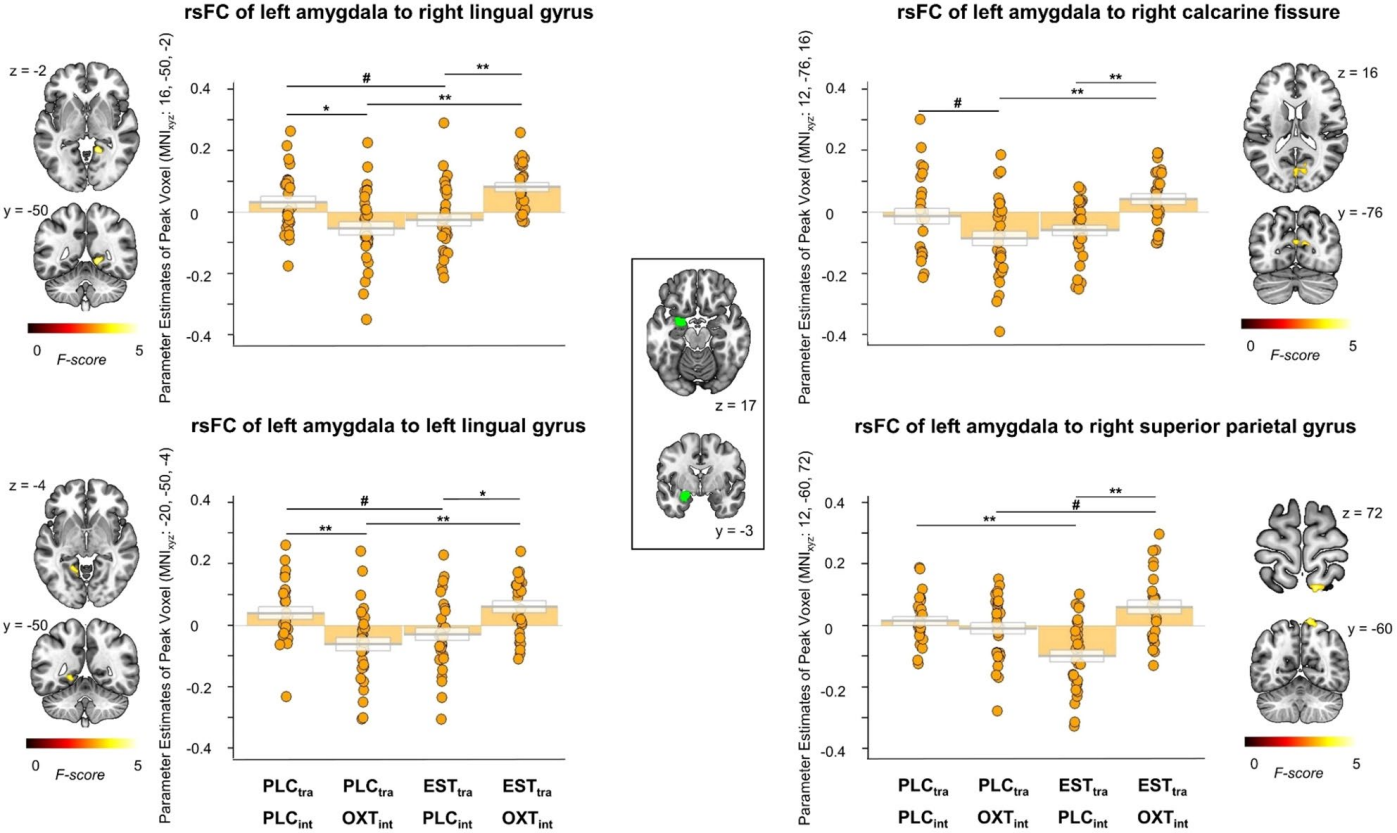
Treatment effects on the resting-state functional connectivity (rsFC) between the left amygdala as seed region (green cluster) and the right and left lingual gyrus, the right calcarine fissure, and the right superior parietal gyrus in men*.* The single treatment with either estradiol or oxytocin significantly decreased rsFC, while the combined treatment led to an rsFC comparable to that of the placebo group. Error bars indicate standard errors of the mean. PLC_tra_ = transdermal placebo gel; PLC_int_ = intranasal placebo; OXT_int_ = intranasal oxytocin; EST_tra_ = transdermal estradiol. #*p* < 0.1, *p < 0.05, **p < 0.01.

To disentangle the observed three-way interactions, we further examined treatment effects separately for each sex. In women, a significant EST_tra_ treatment * OXT_int_ treatment interaction was identified in rsFC of the right hippocampus with the left anterior cingulate gyrus (MNI_xyz_: - 4, 38, 26, *k* = 92, *p*_FDR_ = 0.04; see Fig. 1). By contrast, the interaction effects on amygdala rsFC were evident in men, but not significant in women (see Fig. 2). In men, we found significant interactions between the two treatments in rsFC of the left amygdala with the right and left lingual gyrus (right: MNI_xyz_: 16, - 50, - 2, *k* = 202, *p*_FDR_ < 0.001; left: MNI_xyz_: - 20, - 50, - 4, *k* = 102, *p*_FDR_ = 0.005), the right calcarine fissure (MNI_xyz_: 12, - 76, 16, *k* = 107, *p*_FDR_ = 0.005), and the right superior parietal gyrus (MNI_xyz_: 12, - 60, 72, *k* = 77, *p*_FDR_ = 0.02). Further analyses of the amygdala subregions again showed a significant two-way interaction specifically in rsFC of the left centromedial amygdala with the left rolandic operculum (MNI_xyz_: - 54, 2, 14, *k* = 91, *p*_FDR_ = 0.03) in the male subsample, but not in the female subsample.

Interestingly, we also observed significant two-way interaction effects on rsFC of the DMN in men. Specifically, interaction effects were identified in rsFc of the DMN with the left supramarginal gyrus (MNI_xyz_: 54, - 24, 26, *k* = 130, *p*_FDR_ = 0.003), the right and left superior dorsolateral frontal gyrus (right: MNI_xyz_: 16, 38, - 18, *k* = 123, *p*_FDR_ = 0.003; left: MNI_xyz_: - 16, 34, - 22, *k* = 120, *p*_FDR_ = 0.004), and the left cerebellum Crus 1 (MNI_xyz_: - 32, - 74, - 26, *k* = 130, *p*_FDR_ = 0.003).

To further analyze the significant two-way interactions, we extracted the parameter estimates of the significant peak voxels and employed t-tests to compare the activation between the treatment groups.

#### Treatment effects in women

Analyses of the extracted parameter estimates revealed that EST_tra_ significantly increased rsFC between the right hippocampus and the left anterior cingulate gyrus after PLC_int_ treatment (EST_tra_ & PLC_int_ > PLC_tra_ & PLC_int_: *t*_(51)_ = 4.40, *p*_cor_ < 0.001, *d* = 1.21), but significantly reduced the rsFC in women who received OXT_int_ (EST_tra_ & OXT_int_ < PLC_tra_ & OXT_int_: *t*_(56)_ = - 2.13, *p*_cor_ = 0.04, *d* = - 0.56). Likewise, OXT_int_ significantly enhanced rsFC between the right hippocampus and the left anterior cingulate gyrus after PLC_tra_ treatment (PLC_tra_ & OXT_int_ > PLC_tra_ & PLC_int_: *t*_(57)_ = 3.66*, p*_cor_ < 0.01, *d* = 0.96), but decreased rsFC in women who received EST_tra_ (EST_tra_ & OXT_int_ < EST_tra_ & PLC_int_:* t*_(50)_ = - 3.08, *p*_cor_ < 0.01, *d* = - 0.85).

#### Treatment effects in men

After the PLC_int_ treatment, EST_tra_ decreased rsFC between the left amygdala and the right and left lingual gyrus (EST_tra_ & PLC_int_ < PLC_tra_ & PLC_int_: right: *t*_(57)_ = - 2.03*, p*_cor_ = 0.047, *d* = - 0.53; left: *t*_(57)_ = - 2.38, *p*_cor_ = 0.02, *d* = - 0.62) and the right superior parietal gyrus (EST_tra_ & PLC_int_ < PLC_tra_ & PLC_int_: *t*_(57)_ = - 4.42, *p*_cor_ < 0.001, *d* = - 1.15). The opposite effect was evident in the OXT_int_ treatment groups, with EST_tra_ increasing rsFC between the left amygdala and the right and left lingual gyrus, the right calcarine fissure, and the right superior parietal gyrus (EST_tra_ & OXT_int_ > PLC_tra_ & OXT_int_: right lingual gyrus: *t*_(55)_ = 4.91, *p*_cor_ < 0.001, *d* = 1.31; left lingual gyrus: *t*_(55)_ = 4.14, *p*_cor_ < 0.001, *d* = 1.10; right calcarine fissure: *t*_(55)_ = 4.14, *p*_cor_ < 0.001, *d* = 1.10; right superior parietal gyrus: *t*_(55)_ = 2.46, *p*_cor_ = 0.03, *d* = 0.65). The single treatment with OXT_int_ produced similar effects as the single treatment with EST_tra_. OXT_int_ decreased rsFC between the left amygdala and the right and left lingual gyrus (PLC_tra_ & OXT_int_ < PLC_tra_ & PLC_int_: right: *t*_(56)_ = - 2.89, *p*_cor_ = 0.01, *d* = - 0.76; left: *t*_(56)_ = - 3.35, *p*_cor_ < 0.01, *d* = - 0.88). However, after the EST_tra_ treatment, OXT_int_ increased rsFC (EST_tra_ & OXT_int_ > EST_tra_ & PLC_int_: right lingual gyrus: *t*_(56)_ = 4.08*, p*_cor_ < 0.001, *d* = 1.08; right calcarine fissure: *t*_(55)_ = 4.12, *p*_cor_ < 0.001, *d* = 1.10; left lingual gyrus: *t*_(56)_ = 3.18, *p*_cor_ < 0.01, *d* = 0.84; right superior parietal gyrus: *t*_(56)_ = 5.38, *p*_cor_ < 0.001, *d* = 1.42). The same pattern of results was also evident for parameter estimates of rsFC of amygdala subregions, and DMN (cf. Supplementary Information). Taken together, both single treatments with EST_tra_ and OXT_int_ produced sex-specific effects on rsFC which were reversed in the combined treatment group.

### Neuroendocrine parameters

We collected blood samples before the treatments and after the fMRI (approx. 4.5 h after gel administration) to measure the concentrations of the hormones EST, OXT, testosterone, and progesterone. At baseline, women had significantly higher EST concentrations (*t*_(157.61)_ = 7.29, *p* < 0.001, *d* = 1.00), but lower testosterone (*t*_(114.65)_ = - 28.50, *p* < 0.001, *d* = - 3.71) and OXT levels (t_(221)_ = 2.90, *p* < 0.01, *d* = - 0.39) than men. The progesterone baseline concentrations were comparable between the two sexes (*t*_(107.21)_ = - 1.60, *p* = 0.11, *d* = - 0.22). Importantly, all baseline levels were comparable between treatment groups (all *p*s > 0.05).

The EST_tra_ administration significantly increased blood EST levels in both sexes (see Supplementary Table S1; time * EST_tra_ treatment: *F*_(1, 206)_ = 303.10, *p* < 0.001, η_p_^2^ = 0.60), with women exhibiting a significantly larger increase than men (time * sex * EST_tra_ treatment: *F*_(1, 206)_ = 13.87, *p* < 0.001, η_p_^2^ = 0.06). There were no significant main or interaction effects of the OXT_int_ treatment on EST levels (all *p*s > 0.05), indicating that the OXT_int_ treatment did not modulate the EST increase.

As an additional control analysis, we used a median-dichotomization and excluded EST_tra_-treated women with large EST increase. In this subsample, the treatment-induced increases in EST levels were comparable between women and men within the treatment groups (all *p*s > 0.05) and the rsFC analyses yielded a similar pattern of results (see SI).

OXT_int_ administration significantly increased blood oxytocin levels in both sexes (see Supplementary Table S2; time * OXT_int_ treatment: *F*_(1,213)_ = 347.92, *p* < 0.001, η_p_^2^ = 0.52)*.* There were no significant interaction effects of OXT_int_ and sex on the OXT levels, as well as no significant main or interaction effects of the EST_tra_ treatment on the OXT levels (all *p*s > 0.05), indicating that the EST_tra_ treatment did not modulate the OXT increase.

To examine whether the OXT and EST baseline values, as well as changes in OXT and EST levels affected neural treatment effects, we included the baseline values and the hormonal changes (after treatment minus baseline) as separate covariates in the analyses and all observed sex * treatment interactions remained significant.

## Discussion

The goal of the current study was to elucidate the effects of exogenous EST_tra_ and OXT_int_ treatments and their interaction on hippocampus and amygdala rsFC in healthy women and men. Our results show significant interactions of sex and the treatments on hippocampus and amygdala rsFC. In women, the single treatment with either EST_tra_ or OXT_int_ significantly increased rsFC between the right hippocampus and the left anterior cingulate gyrus, while the combined treatment had the opposite effect. In men, both hormones significantly decreased the rsFC between the left amygdala and the right and left lingual gyrus, the right calcarine fissure, and the right superior parietal gyrus. Again, the combined treatment produced the opposite effect. Collectively, our study indicates an essential role of EST_tra_ and OXT_int_ as modulators of rsFC. We observed sex-specific differences in the localization of the rsFC effects, but the combined treatment reversed the single treatment effects in both sexes and produced effects comparable to the placebo groups, indicating an antagonistic effect of the two hormones at the administered doses.

While some studies found no significant sex differences in rsFC^69^, recent machine learning approaches were able to reliably classify sex based on sex specifics in the functional brain organization both within sample and across independent samples^55^. Specifically, there is accumulating evidence that women and men differ in the rsFC of the amygdala^62–64^ and hippocampus^64,65^. However, the direction of these changes (e.g. increased or decreased functional connectivity) varies between studies. In contrast to our hypothesis that women and men would differ in their amygdala and hippocampus rsFC under placebo^62–65^, our results only showed a decreased rsFC between the right hippocampus and the left anterior cingulate cortex (ACC) in women compared to men, but no sex difference in rsFC of the amygdala. However, previously observed sex differences appear to depend on methodological details such as analysis method or sample characteristics. In the current study, we exclusively tested free-cycling women in their early follicular phase, which is associated with low levels of fluctuating steroid hormones, and excluded women using oral contraceptives. In contrast, previous sex-specific rsFC effects were reported in studies involving women in different cycle phases^63^, and in studies not focusing on cycle phase or the use of oral contraceptives^62,64,65^. Thus, previously observed sex differences in rsFC of the amygdala might reflect the impact of other sex steroids fluctuating across the menstrual cycle, such as progesterone, or the influence of oral contraceptives on rsFC^70,71^.

In our study, the single treatment with EST_tra_ did not significantly affect amygdala rsFC in women. Our hypothesis that EST_tra_ would affect amygdala rsFC in women was based on a previous study^46^, which showed an association between increased amygdala rsFC and higher estradiol levels. We used an exogenous administration of estradiol to selectively modulate EST levels in healthy women in the early follicular phase. Thus, natural fluctuations of other hormones may have contributed to the previously observed association with estradiol. However, in line with our hypothesis, we observed that EST_tra_ increased hippocampal rsFC to the left ACC in women. The effect of estrogens on the hippocampus has been often investigated in female rodents^72^ and most of the studies demonstrated a positive effect of estrogens on the hippocampal neurogenesis and dendritic morphology. Previous rsFC research on effects of EST on hippocampal connectivity revealed prefrontal regions^45^, but also in line with our results the ACC as key target region^73–75^. Following a treatment with a gonadotropin releasing hormone agonist, which caused reduced EST levels, the functional connectivity between the hippocampus and the ACC was decreased^73^. In another study, postpartum women, who experience a sudden decline in EST levels after birth, demonstrated a decrease in rsFC of the hippocampus to the ACC^74,75^. Estrogen-dependent modulations of the hippocampus morphology and activation have been mostly examined in women and studies on EST effects in men are scarce. In our study, a single EST_tra_ treatment produced no significant effect in the male hippocampal rsFC. As yet, no study probed the effects of exogenous EST on rsFC in men. There is some evidence that changes in rsFC of the hippocampus depend on hippocampal neurogenesis in female mice^76^. However, previous studies^77,78^ on hippocampal neurogenesis did not detect significant EST effects in male rats, either, and suggest an androgen-dependent mechanism, but it is unclear whether and how altered neurogenesis translates to altered rsFC in humans.

The single OXT_int_ treatment significantly increased hippocampal rsFC to the left ACC in women. Most published work on OXT_int_ effects focuses exclusively on males, but previous studies on OXT_int_ effects in women did not report significant modulation of hippocampus rsFC following an OXT_int_ treatment^18,32,66^. These differences might be rooted in experimental differences, as two of the studies examined women in different cycle phases^32,66^ or additionally used a higher OXT dosage^66^. While Bethlehem and colleagues^18^ only included women in their early follicular phase of their menstrual cycle and used the same dosage as in this study, they applied an independent component analysis (ICA) to examine how connectivity between-circuits differ across placebo and OXT_int_. Based on our hypotheses about rsFC of the hippocampus and amygdala, we employed a seed-to-voxel approach which may produce conceptually different results than ICA^79^. In men, the OXT_int_ treatment significantly decreased left amygdala rsFC to the left and right lingual gyrus compared to the placebo group. While some previous OXT_int_ rsFC studies reported an enhancement of the amygdala rsFC to frontal regions after an OXT_int_ treatment^80^, other studies found a decreased rsFC to the precuneus, prefrontal regions, or the lingual gyrus^33,81,82^. Interestingly, we also observed a significant effect of OXT_int_ on rsFC of the centromedial amygdala, which has been identified as a key target region of possible anxiolytic mechanisms of OXT^34^.

Overall, our results show that a single OXT_int_ treatment yields effects similar to the EST_tra_ treatment, whereas the effects in the combined treatment groups were comparable to the placebo groups. Interestingly, the same pattern of results was evident in our previous study about hippocampus-dependent episodic memory effects of both hormones^53^. This pattern might be the result of an increased OXT receptor binding induced by the EST_tra_ pretreatment^83^, which matches previously observed opposing effects for higher OXT_int_ doses in men^84^. Therefore, the antagonistic interaction of EST_tra_ and OXT_int_ may have contributed to previously observed sex-specific effects of OXT^23,27,85^ and to the modulatory effects of hormonal contraception^86^. However, in contrast to our hypothesis, a pre-treatment with EST_tra_ did not modulate OXT effects on rsFC in the same seed region in women and men. As such, the OXT-EST interactions cannot completely explain the observed sex-specific effects of OXT and future studies are warranted to probe the interaction with other sex hormones like progesterone.

The present study has some limitations that need to be addressed in future research. We tested women during the early follicular phase of their menstrual cycle to control for changes in endogenous hormone levels. Nevertheless, in both sexes, supraphysiological EST levels were induced and it is conceivable that treatment or interaction effects would be altered at physiological EST levels occurring during the menstrual cycle. In addition, the treatment-induced EST levels were higher in women than in men, which may have contributed to the observed sex-specific treatment effects. However, the inclusion of treatment-induced changes in hormonal levels as covariates did not alter our results and importantly, the baseline levels of EST, OXT, testosterone, and progesterone were comparable between treatment groups. Yet, we cannot extrapolate our findings to other cycle phases, which involve the fluctuation of other steroid hormones, or hormonal contraceptives, as different estrogen types and dosages are used for their preparation^87^. Thus, future rsFC studies are warranted to examine possible interactions between endogenous hormones by comparing the effects of experimentally induced release of endogenous OXT (e.g. via synchronous social interactions^84^) between different phases of the menstrual cycle. Future studies on exogenous effects should employ different doses and long-term applications in women and men to further disentangle the impact of exogenous hormones on rsFC.

Collectively, our results provide support for the notion that hippocampus and amygdala rsFC are modulated by sex and by the single and interactive effects of EST_tra_ and OXT_int_. Previous findings associating OXT or EST and rsFC may have been affected by other fluctuating hormones and their potentially interactive effects. Thus, integrating sex and hormonal effects into research designs is vital to further decipher the interaction of neurobiological factors modulating hippocampus and amygdala rsFC.

## Methods

### Ethics and enrolment

The study was part of a larger project (for further results see^53^). It was approved by the institutional review board of the Medical Faculty of the University of Bonn and was carried out in accordance with the latest revision of the Declaration of Helsinki. The study was registered in the ClinicalTrials.gov database (Identifier: NCT04330677) and the data analyses were pre-registered (https://osf.io/gkd6s/). The participants were enrolled in the study after giving informed consent. The participants received monetary reimbursement.

### Participants

In total, 295 participants (160 women) were invited to a screening session prior to the testing session. The 246 participants (122 women) who met the inclusion criteria (see below) were tested. The participants were randomly assigned to one of four experimental conditions: (1. PLC_tra_ & PLC_int_; 2. PLC_tra_ & OXT_int_; 3. EST_tra_ & PLC_int_; 4. EST_tra_ & OXT_int_). The data of 7 participants were excluded due to technical malfunctions. Furthermore, two participants were excluded due to excessive head movements (> 20% volumes were identified as outliers by ART). Additional 9 participants were excluded due to anatomical (n = 2) or hormonal (n = 7) abnormalities and one participant did not finish the study. Thus, after the exclusion of 19 participants from all analyses, our final sample included 227 participants (PLC_tra_ & PLC_int:_ 27 men, 26 women; PLC_tra_ & OXT_int_: 31 men, 33 women; EST_tra_ & PLC_int_: 32 men, 27 women; EST_tra_ & OXT_int_: 26 men, 25 women). For demographic and psychometric baseline characteristics see Supplementary Table S3.

### Screening session and exclusion criteria

The participants were screened in a separate session prior to the test session. The participants were right-handed, non-smoking, and between 18 and 40 years old. Exclusion criteria were MRI contraindications, current pregnancy and the use of hormonal contraceptives. Additionally, participants reported to be free of current or past physical or psychiatric illnesses assessed by the Mini-International Neuropsychiatric Interview^88^ and were naïve to prescription-strength psychoactive medication. Furthermore, participants had not taken any over-the-counter psychoactive medications in the four weeks prior to the study and were asked to abstain from alcohol intake on the day of the experiment. After completing the screening session, the participants were invited to the fMRI testing session. To ensure that the women were tested in their early follicular phase of their menstrual cycle, they were scanned simultaneously with the onset of their menstruation (days 1–6), which was determined via self-report. To further validate the cycle phase, blood assays were obtained on the testing day. Female participants showing estradiol pre-treatment values larger than 145 pg/ml were excluded, because it can be assumed that they were not in the follicular phase of their menstrual cycle^89^.

### Treatments

#### Estradiol/placebo gel treatment

EST_tra_ gel (Estramon, 2 mg EST, Hexal AG, Holzkirchen, Germany) or placebo gel (2 mg ultrasonic gel) was transdermally applied to the participants’ backs. The 2 mg dose was chosen in line with a pharmacokinetic study^90^ to reduce the possibility of side effects. The same dose has also been found to increase emotional vicarious reactivity in men when watching a distressed other^16^.

#### Intranasal oxytocin/placebo treatment

The OXT dosage of 24 International Units (IU) was chosen on the basis of one of our previous studies targeting amygdala functioning^60^, which determined the most effective dose (24 IU, in contrast to 12 IU or 48 IU) and dose-test interval (30–60 min). The participants self-administered 24 IU of synthetic OXT_int_ (Sigma-Tau Industrie Farmaceutiche Riunite S.p.A., Rome, Italy) or placebo via nasal spray prior to the fMRI scanning under supervision of a trained research assistant and in accordance with the latest standardization guidelines^91^. The placebo solution contained identical ingredients as the OXT_int_ solution except for the peptide itself. An interpuff interval of approx. 45 s was chosen and the amount of administered substance was weighed and supplemented until the 24 IU were reached. There is compelling evidence that OXT_int_ bypasses the blood–brain barrier and elevates OXT concentrations in the cerebrospinal fluid^92,93^ and brain^94^.

### Resting state paradigm

Each participant was positioned in the MRI scanner with their heads comfortably placed and stabilized with cushions to reduce head motion. Participants were instructed to relax and to look at a white fixation cross on a black screen for ten minutes.

### Experimental design

We used a randomized, placebo-controlled, double-blind, parallel-group study design. The fMRI day commenced with the gel administration. The OXT_int_ or placebo spray was administered three hours after gel administration in line with our pharmacokinetic pre-study (see **SI**). The imaging data collection included a high-resolution structural MRI scan and a resting-state scan, followed by two tasks (for further results see^53^). The resting-state data collection commenced 35 min after OXT_int_ administration, because the strongest limbic effects can be expected for dose-test interval of 30–60 min^60^. Blood samples were collected at baseline and immediately after the fMRI testing session (approx. 4.5 h after gel administration).

### Data analysis

#### fMRI data acquisition

All fMRI data were acquired using a 3 T Siemens TRIO MRI system (Siemens AG, Erlangen, Germany) with a Siemens 32-channel head coil. Following a fieldmap acquisition, resting state data were acquired using T2*-weighted echoplanar (EPI) sequence [repetition time (TR) = 2690 ms, echo time (TE) = 30 ms, ascending slicing, matrix size: 96 × 96, voxel size: 2 × 2 × 3 mm^3^, slice thickness = 3.0 mm, distance factor = 10%, FoV = 192 mm, flip angle 90°, 41 axial slices] for ten minutes. High-resolution T1-weighted structural images were collected on the same scanner (TR = 1660 ms, TE = 2.54 ms, matrix size: 256 × 256, voxel size: 0.8 × 0.8 × 0.8 mm^3^, slice thickness = 0.8 mm, FoV = 256 mm, flip angle = 9°, 208 sagittal slices).

#### fMRI data preprocessing

The resting state data was analyzed employing the Functional Connectivity Toolbox for SPM (CONN; http://www.nitrc.org/projects/conn/)^95^. The CONN preprocessing pipeline included realignment and unwarping using the fieldmap, slice time correction, segmentation, spatial normalization, and smoothing with a 6 mm Gaussian kernel. Furthermore, to limit the impact of head movements, the artifact detection tool (ART) implemented in CONN was used to identify high motion volumes using a volume-to-volume shift of  > 1.5 mm and a volume-to-volume change in mean signal intensity of  > 3 standard deviations. Artifacts were treated as regressors of no interest in the following analysis. Participants with > 20% volumes identified as outliers by ART were excluded.

#### fMRI data analysis

Amygdalar and hippocampal functional resting-state connectivity was probed in a whole brain seed-to-voxel analysis. To assess the connectivity of the amygdala and hippocampus, seed-to-voxel connectivity maps were estimated for each participant using CONN. The seeds (left and right hippocampus and left and right amygdala) were anatomically defined using the aal atlas in the Wake Forest University PickAtlas, version 3.0. Statistical analyses were conducted using CONN. Connectivity maps for each of the seeds were compared using analyses of variance (ANOVAs) with the between-subjects variables “nasal spray treatment” (PLC_int_ or OXT_int_), “gel treatment” (EST_tra_ or PLC_tra_), and “sex” (female or male). We probed significant three-way interactions and two-way interactions separately for men and women. Parameter estimates of significant peak voxels of the significant two-way interactions (cluster defining threshold *p* < 0.001; significance threshold *p* < 0.05, false discovery rate-corrected, *p*_FDR_) were extracted and further analyzed with SPSS 27 (IBM Corp., Armonk, NY). Post-hoc analyses employed two-sample t-tests comparing activation between subgroups, corrected for multiple comparisons with the Bonferroni-Holm method (*p*_cor_).

### Further exploratory analyses

We explored the rsFC of amygdala subregions, and the DMN with whole brain seed-to-voxel analyses. The basolateral, centromedial, and superficial amygdala were defined as seeds based on cytoarchitectonic probabilistic maps^96^ implemented in the Anatomy toolbox^97^. The four DMN main nodes (medial prefrontal cortex (MPFC), left and right lateral parietal regions, and the posterior cingulate cortex (PCC)) were based on the pre-defined seeds implemented in the CONN toolbox. We employed the four seeds separately to detect possible differences in the resulting connectivity maps. The preprocessing and the data analysis of the amygdala subregions, and the DMN were the same as described for the amygdala and hippocampus whole brain seed-to-voxel analysis.

### Statistical analyses

Neuroendocrine and demographic data were analyzed in SPSS 27 using standard procedures including analyses of variances (ANOVAs) and post-hoc *t*-tests. Post hoc *t*-tests were Bonferroni-Holm-corrected (*p*_cor_). If the assumption of sphericity was significantly violated, a Greenhouse–Geisser correction was applied. As measures of effect sizes, partial eta-squared and Cohen’s d were calculated. Changes in hormone concentrations were examined with mixed-design ANOVAs with the between-subject factors “OXT_int_ treatment”, “EST_tra_ treatment”, and “sex” and the within-subject variable “time” (baseline, after fMRI). Furthermore, to explore the potential moderating effects of treatment-induced hormonal changes, the magnitude of the increases in hormone concentrations (levels of EST, OXT, testosterone, and progesterone after the fMRI session minus baseline) as well as autistic-like traits and social anxiety scores were considered covariates in the main analyses with significant neural outcomes (i.e., parameter estimates of significant contrasts of interests).

### Ethical approval

This study protocol was reviewed and approved by the institutional review board of the medical faculty of the University of Bonn [Approval number: 213/16]. Written informed consent was obtained from all participants included in this study.

## Supplementary Information

Supplementary Information.

## Data Availability

The data that support the findings of the present study are openly available in the repository of the Open Science Foundation at https://osf.io/xubts/ (https://doi.org/10.17605/OSF.IO/XUBTS). The code that supports the findings of the present study is openly available in the repository of the Open Science Foundation at https://osf.io/xubts/ (https://doi.org/10.17605/OSF.IO/XUBTS).
