# Supplementary material for: Effects of exogenous oxytocin and estradiol on resting-state functional connectivity in women and men
Source: Sci Rep. 2023 Feb 22;13:3113. doi: 10.1038/s41598-023-29754-y (PMC9947123; doi:10.1038/s41598-023-29754-y)
Supplement: Supplementary file 1 — Supplementary Information. [file 41598_2023_29754_MOESM1_ESM.docx]

**Supporting Information**

Effects of exogenous oxytocin and estradiol on resting-state functional connectivity in women and men

Marie Coenjaerts, Berina Adrovic, Isabelle Trimborn, Alexandra Philipsen, René Hurlemann, Dirk Scheele

**Supplementary Methods**

As the study was part of a larger project, methods and results concerning the pharmacokinetic pre-study, power analysis, neuroendocrine parameters, side effects, blinding of treatment, body mass index and hormonal levels, missing values, demographic and psychometric baseline characteristics, and measurements of mood were already described in our previous paper^[1]^, but adapted for the current sample.

**Pharmacokinetic pre-study**

We conducted a prestudy involving ten healthy participants (5 women; mean age ± SD = 24.10 ± 4.07 years) to examine the pharmacokinetics of estradiol (EST) gel (Estramon 2 mg estradiol, Hexal AG, Holzkirchen, Germany) administration. Blood samples were taken prior to EST administration (i.e. baseline) and in 1-hour intervals after drug application up to five hours post administration. An additional blood sample was taken the next day (after 18 hours). Serum EST levels peaked 3-4 hours after gel administration, but a significant increase relative to baseline was already evident after two hours (*t*_(9)_ = 2.44, *p* = 0.04, *d* = 1.10; **cf.^[2]^**). EST levels remained significantly elevated throughout the last measurement. A previous study tested the topical administration of a different drug (Divigel, Orion Pharma AG, Zug, Switzerland) containing 2 mg EST and found significantly increased EST serum concentrations as soon as one hour after administration and maximum average levels after two hours^[3]^.

**Power analysis**

This study was part of a larger project and we used G*Power 3^[4]^ to conduct an a-priori power analysis for the project. We based the power analysis on the effect size obtained in our OXT dose-response study^[5]^. Regarding the effect of intranasal oxytocin (OXT, 24 IU at a latency of 45 minutes) on the amygdala response to high intensity fearful faces we observed an effect size of *dz* = 0.56 in a within-subject design. To detect an OXT effect of this size (with α = 0.05 and power = 0.75), we needed to test at least 48 participants in a between-subject design (i.e. 24 male participants in the placebo group (PLC) and 24 male participants in the OXT group). Thus, we planned to include at least 24 participants in each treatment group (1. PLC & PLC; 2. PLC & OXT; 3. EST & PLC; 4. EST & OXT) separately for both sexes (1. female; 2. male). In total, 122 healthy women and 124 healthy men were included in the study to control for drop-outs and exclusions. The final sample for the analyses included 53 participants (26 women) in the PLC_tra_ and PLC_int_ group, 64 participants (33 women) in the PLC_tra_ and OXT_int_ group, 59 participants (27 women) in the EST_tra_ and PLC_int_ group, and 51 participants (25 women) in the EST_tra_ and OXT_int_ group.

**Neuroendocrine parameters**

*EST and other gonadal hormones*

Serum EST, testosterone and progesterone were determined by fully automated electrochemiluminescent immunoassays (ECLIA, Elecsys test) on a cobas e801 analyzer (Roche Diagnostics, Mannheim, Germany) according to the manufacturer’s instructions (Roche Diagnostics). The coefficients of variation for intra-assay and inter-assay precision were 1.63% and 2.51% for EST, 2.27% and 3.71% for testosterone, and 2.28 % and 2.83 % for progesterone, respectively.

*Oxytocin*

Plasma samples for the measurement of OXT concentrations were collected with commercial sampling devices (Vacuette, Greiner Bio-One International, Austria) containing ethylenediaminetetraacetic acid (EDTA) and aprotinin. Vacuettes were immediately centrifuged at 3250 rpm for ten minutes, and aliquoted samples were stored at -80°C until assayed. OXT concentrations were extracted and quantified using a highly sensitive and specific radioimmunoassay (RIAgnosis, Munich, Germany). The limit of detection was 0.1-0.5 pg, depending on the age of the tracer. Intra-assay and inter-assay coefficients of variability were <10%. All samples to be compared were assayed in the same batch, i.e. under intra-assay conditions.

**Questionnaires**

To characterize the sample, we assessed depressive symptoms (Becks Depression Inventory [BDI]^[6]^), social anxiety (Liebowitz Social Anxiety Scale [LSAS]^[7]^), autistic-like traits (Autism Spectrum Quotient [AQ]^[8]^) and trait anxiety (State Trait Anxiety Inventory [STAI]^[9]^). We used Qualtrics software for the presentation of the questionnaires (Provo, USA).

**Supplementary Results**

***Additional treatment effects in men***

To further analyze the significant two-way interactions of the amygdala subregions, and the DMN, we extracted the parameter estimates of the significant peak voxels and employed t-tests to compare the activation between the treatment groups. Analyses of the amygdala subregions showed that under PLC_int_, EST_tra_ reduced the rsFC between the left centromedial amygdala and the left rolandic operculum (EST_tra_ & PLC_int_ < PLC_tra_ & PLC_int_: *t*_(57)_ = -3.36*, p*_cor_ < 0.01, *d* = -0.88), whereas the direction of the effect was reversed after the OXT_int_ treatment (EST_tra_ & OXT_int_ > PLC_tra_ & OXT_int_: *t*_(55)_ = 4.38*, p*_cor_ < 0.01, *d* = 1.17). Likewise, OXT_int_ decreased the rsFC between the left centromedial amygdala and the left rolandic operculum after PLC_tra_ treatment (PLC_tra_ & OXT_int_ < PLC_tra_ & PLC_int_: *t*_(56)_ = -4.51*, p*_cor_ < 0.01, *d* = -1.19), but significantly increased rsFC after the EST_tra_ treatment (EST_tra_ & OXT_int_ > EST_tra_ & PLC_int_: *t*_(56)_ = 3.19*, p*_cor_ < 0.01, *d* = 0.84).

Further analyses of the extracted parameter estimates revealed that after the PLC_int_ treatment, EST_tra_ decreased rsFC between the DMN and the right and left superior dorsolateral frontal gyrus (EST_tra_ & PLC_int_ < PLC_tra_ & PLC_int_: right: *t*_(57)_ = -3.38*, p*_cor_ < 0.01, *d* = -0.88; left: *t*_(57)_ = -3.13*, p*_cor_ = 0.01, *d* = -0.82) and the left Cerebellum Crus 1 (EST_tra_ & PLC_int_ < PLC_tra_ & PLC_int_: *t*_(57)_ = -4.13*, p*_cor_ < 0.01, *d* = -1.08). However, combined with the OXT_int_ treatment, EST_tra_ increased the rsFC between the DMN and the right and left superior dorsolateral frontal gyrus (EST_tra_ & OXT_int_ > PLC_tra_ & OXT_int_: right: *t*_(55)_ = 4.30*, p*_cor_ < 0.01, *d* = 1.14; left: *t*_(55)_ = 4.17*, p*_cor_ < 0.01, *d* = 1.11) and the left Cerebellum Crus 1 (EST_tra_ & OXT_int_ > PLC_tra_ & OXT_int_: *t*_(55)_ = 2.56*, p*_cor_ = 0.02, *d* = 0.68), but reduced rsFC with the right supramarginal gyrus (EST_tra_ & OXT_int_ < PLC_tra_ & OXT_int_: *t*_(55)_ = -4.98*, p*_cor_ < 0.01, *d* = -1.33). After the PLC_tra_ treatment, OXT_int_ decreased rsFC between the DMN and the right and left superior dorsolateral frontal gyrus (PLC_tra_ & OXT_int_ < PLC_tra_ & PLC_int_: right: *t*_(56)_ = -3.33*, p*_cor_ < 0.01, *d* = -0.88; left: *t*_(56)_ = -3.94*, p*_cor_ < 0.01, *d* = -1.04) and the left Cerebellum Crus 1 (PLC_tra_ & OXT_int_ < PLC_tra_ & PLC_int_: *t*_(56)_ = -3.09*, p*_cor_ = 0.02, *d* = -0.81), but increased the rsFC with the right supramarginal gyrus (PLC_tra_ & OXT_int_ > PLC_tra_ & PLC_int_: *t*_(56)_ = 3.94*, p*_cor_ < 0.01, *d* = 1.04). Again, this pattern was reversed after the combined treatment with EST_tra_ (EST_tra_ & OXT_int_ > EST_tra_ & PLC_int_: right superior dorsolateral frontal gyrus: *t*_(56)_ = 4.33*, p*_cor_ < 0.01, *d* = 1.14; left superior dorsolateral frontal gyrus: *t*_(56)_ = 3.39*, p*_cor_ < 0.01, *d* = 0.90; left Cerebellum Crus 1: *t*_(56)_ = 3.61*, p*_cor_ < 0.01, *d* = 0.95; EST_tra_ & OXT_int_ < EST_tra_ & PLC_int_: right supramarginal gyrus: *t*_(56)_ = -3.02*, p*_cor_ = 0.02, *d* = -0.80).

**Supraphysiological EST levels**

Women exhibited a significantly larger increase in blood EST levels than men. To address that difference, we included the EST increase as a covariate in our analyses. To further probe the impact of this difference, we used a median-dichotomization and excluded EST_tra_-treated women with large EST increase (n = 26). In the remaining subsample (n = 200), the treatment-induced increases in EST levels were comparable between women and men within the treatment groups (all *p*s > 0.05). We repeated the main analyses with the parameter estimates of the significant three-way interactions with this subsample and observed a similar pattern of results. In line with our reported main findings, the significant three-way interactions identified in rsFC of the right hippocampus and the left anterior cingulate gyrus remained significant in this subsample (*F*_(1, 192)_ = 21.50, *p* < 0.001, η_p_^2^ = 0.10). Further analyses revealed that the significant three-way interactions, which were observed in rsFC of the left amygdala and the right lingual gyrus and the left cuneus remained significant (right lingual gyrus: *F*_(1, 193)_ = 15.82, *p* < 0.001, η_p_^2^ = 0.08; left cuneus: *F*_(1, 192)_ = 11.48, *p* < 0.01, η_p_^2^ = 0.06). The reported three-way interactions of the rsFC in the amygdala subregions revealed comparable results to our main findings. For the rsFC of the left centromedial amygdala, similar significant three-way interactions with the left cuneus (*F*_(1, 193)_ = 18.08, *p* < 0.001, η_p_^2^ = 0.09), the left lingual gyrus (*F*_(1, 193)_ = 23.63, *p* < 0.001, η_p_^2^ = 0.11), and the right calcarine gyrus (*F*_(1, 193)_ = 19.84, *p* < 0.001, η_p_^2^ = 0.09) were identified. For the right superficial amygdala as a seed region, we still observed a significant three-way interaction with the right frontal lobe (*F*_(1, 193)_ = 20.43, *p* < 0.001, η_p_^2^ = 0.10). Therefore, the significantly larger EST increase in women than men has no major impact on the results.

**Side effects**

Three days following the treatment, the participants were asked to report the side effects of their treatment. No participant experienced severe side effects, but 10.1% of the participants reported side effects consisting of light headache, tiredness, dizziness, sleep disturbance, hilarity, lack of concentration, or circulatory problems. Importantly, the proportion of participants who reported side effects did not significantly differ between the treatment groups (PLC_tra_ & PLC_int_: 13.7%, PLC_tra_ & OXT_int_: 10.0%, EST_tra_ & PLC_int_: 7.0%, EST_tra_ & OXT_int_: 10.0%; *χ*^2^_(3)_ = 1.34, *p* = .72).

**Blinding of treatment**

Both the participants and the experimenters who conducted the experiment were blinded to treatments. Following the MRI scan, the participants were asked to guess which treatment they received. Out of the 115 participants (109 with available estimates) in the OXT groups (PLC_tra_ & OXT_int_ and EST_tra_ & OXT_int_ ), 27 (24.8%; 11 men) believed that they had received OXT, while 25 (23.4%; 12 men) subjects in the PLC groups (PLC_tra_ & PLC_int_ and EST_tra_ & PLC_int_; *n* = 112, 107 with available estimates) believed that they had received verum treatment. Out of the 110 participants in the EST groups (EST_tra_ & PLC_int_ and EST_tra_ & OXT_int_ 108 with available estimates), 23 (21.3%; 13 men) estimated that they had received verum treatment, while 22 (20.4%; 4 men) subjects in the PLC groups (PLC_tra_ & PLC_int_ and PLC_tra_ & OXT_int;_ *n* = 117, 108 with available estimates) assumed that they had received EST.

In general, the participants who believed in an OXT treatment also guessed an EST treatment (*r*_(214)_ = 0.41, *p* < 0.001). However, the treatment estimates were not significantly associated with the actual treatments (all *p*s > 0.05).

**Body mass index and hormonal levels**

The body mass index (BMI) of the participants did not significantly correlate with the baseline EST or OXT levels in men or women (all *p*s > 0.05) or with treatment-induced EST_tra_ increases in either sex (all *p*s > 0.05). Thus, the EST_tra_ treatment did not result in different peripheral levels depending on BMI. While OXT_int_ treatment did not produce different peripheral OXT levels depending on the BMI in men (*p* > 0.05), the BMI positively correlated with the OXT increase in OXT_int_-treated women (BMI: *r*_(53)_ = 0.33, *p* = 0.02). Thus, OXT_int_ treatment resulted in higher peripheral OXT levels in women with a higher BMI.

**Missing values**

The following blood samples were missing due to problems in sample assessment or analysis: baseline (OXT, n = 4; EST, n = 6; progesterone, n = 6; testosterone, n = 7), post-treatment (OXT, n = 2; EST, n = 7; progesterone, n = 3; testosterone, n = 9), and three days after the treatment (EST, n = 15; progesterone, n = 12; testosterone, n = 15). Connection issues and technical errors resulted in the loss of questionnaires evaluating depressive symptoms (n = 9), autistic-like traits (n = 4), alexithymia (n = 4), trait anxiety (n = 5), social anxiety (n = 4), treatment guesses (n = 11), pretreatment negative and positive mood (n = 3), and posttreatment negative and positive mood (n = 10).

**Demographic and psychometric baseline characteristics**

Demographics and baseline psychometric assessments of the participants are displayed in **Table S3**. There were no significant differences in age, alexithymia, depressive symptoms, social anxiety, and trait anxiety between treatment groups within sexes (all *p*s_cor_ > 0.05). In men, there were no significant differences in the autistic-like traits between treatment groups, in contrast to the women (main effect of treatment group: *F*_(3, 104)_ = 117.94, *p* = 0.01, η_p_^2^ = 0.10). Post-hoc tests revealed that women in the combined treatment group and in the PLC treated group reported higher autistic like traits than the OXT_int_-treated women (PLC_tra_ & OXT_int_ < EST_tra_ & OXT_int_: *t*_(33.06)_ = 2.89*, p*_cor_ = 0.03, *d* = 0.83; PLC_tra_ & OXT_int_ < PLC_tra_ & PLC_int_: *t*_(33.80)_ = 2.49*, p*_cor_ = 0.03, *d* = 0.71), while the other treatment groups were not significantly different. Across treatments women reported significantly higher social anxiety (Liebowitz scale, *t*_(221)_ = -4.18 , *p* < 0.001, *d* = -0.56) and increased trait anxiety (STAI Trait*, t*_(220)_ = -3.17, *p* < 0.01, *d* = -0.43) than men. Age, alexithymia, depressive symptoms, and autistic-like traits were not significantly different between the sexes (all *ps* > 0.05). Autistic-like traits and social anxiety were included as covariates in the main analyses of the significant neural main results (hippocampal and amygdala three- and two-way interactions). All reported effects remained significant and the covariates showed no significant effect (all *ps* > 0.05).

**Measurements of mood**

A main effect of time was found for the positive (*F*_(1,206)_ = 92.98, *p* < 0.001, η_p_^2^ = .31) and negative (*F*_(1,206)_ = 9.23, *p* < 0.01, η_p_^2^ = .04) affect measured with the PANAS (the Positive and Negative Affect Schedule) for both sexes (cf. **Table S4**). Positive and negative mood significantly decreased from the beginning of the testing session (mean ± SD positive: 28.84 ± 6.03; negative: 12.15 ± 3.43) to the end (mean ± SD positive: 25.15 ± 6.50; negative: 11.55 ± 2.19), suggesting an increasing fatigue over the time course of the experiment. In addition, we observed a significant main effect of sex for the positive mood. Men reported significantly higher positive mood than women (beginning: *t*_(222)_ = 2.18, *p =* 0.03, *d* = 0.29; end: *t*_(215)_ = 3.36, *p* = 0.001, *d* = 0.46) No further significant main or interaction effects of time and EST_tra_ and OXT_int_ treatments were found for positive or negative mood.

**References**

1. Coenjaerts, M. *et al.*, Exogenous estradiol and oxytocin modulate sex differences in hippocampal reactivity during the encoding of episodic memories. *NeuroImage*, 119689 (2022).

2. Coenjaerts, M. *et al.*, Sex differences in economic decision-making: Exogenous estradiol has opposing effects on fairness framing in women and men. *Eur Neuropsychopharmacol* **50**, 46-54 (2021).

3. Eisenegger, C., von Eckardstein, A., Fehr, E., von Eckardstein, S., Pharmacokinetics of testosterone and estradiol gel preparations in healthy young men. *Psychoneuroendocrinology* **38**, 171-178 (2013).

4. Faul, F., Erdfelder, E., Lang, A. G., Buchner, A., G*Power 3: a flexible statistical power analysis program for the social, behavioral, and biomedical sciences. *Behav Res Methods* **39**, 175-191 (2007).

5. Spengler, F. B. *et al.*, Kinetics and Dose Dependency of Intranasal Oxytocin Effects on Amygdala Reactivity. *Biol Psychiatry* **82**, 885-894 (2017).

6. Beck, A. T., Steer, R. A., Brown, G. K., Beck depression inventory-II. *Psychological assessment*, (1996).

7. Liebowitz, M. R., Social phobia. *Mod Probl Pharmacopsychiatry* **22**, 141-173 (1987).

8. Baron-Cohen, S., Hoekstra, R. A., Knickmeyer, R., Wheelwright, S., The Autism-Spectrum Quotient (AQ)--adolescent version. *J Autism Dev Disord* **36**, 343-350 (2006).

9. Spielberger, C. D., Manual for the state-trait anxietry, inventory. *Consulting Psychologist*, (1970).

**Table S1.** Estradiol, progesterone and testosterone concentrations at baseline, immediately post treatment and three days after the treatment

|  |  | Females | | | | Males | | | |
| --- | --- | --- | --- | --- | --- | --- | --- | --- | --- |
|  |  | PLC_tra_ & PLC_int_  Mean  (n, ± SD) | PLC_tra_ & OXT_int_  Mean  (n, ± SD) | EST_tra_ & PLC_int_  Mean  (n, ± SD) | EST_tra_ & OXT_int_  Mean  (n, ± SD) | PLC_tra_ & PLC_int_  Mean  (n, ± SD) | PLC_tra_ & OXT_int_  Mean  (n, ± SD) | EST_tra_ & PLC_int_  Mean  (n, ± SD) | EST_tra_ & OXT_int_  Mean  (n, ± SD) |
| Estradiol  (pg/ml) | pre | 34.80  (26, 19.44) | 36.86  (32, 17.94) | 45.98  (25, 25.96) | 38.51  (24, 16.90) | 22.39  (26, 7.86) | 25.83  (31, 11.54) | 21.90  (32, 11.73) | 20.52  (25, 10.33) |
|  | post | 40.72  (25, 19.31) | 41.44  (32, 17.33) | 985.85  (27, 576.68) | 990.96  (24, 445.67) | 26.88  (27, 10.71) | 31.92  (30, 10.36) | 587.03  (31, 440.82) | 662.38  (24, 419.42) |
| Progesterone  (ng/ml) | pre | 1.01  (26, 3.47) | 0.26  (32, 0.24) | 0.29  (25, 0.27) | 0.19  (24, 0.11) | 0.16  (26, 0.17) | 0.22  (31, 0.14) | 0.15  (32, 0.11) | 0.13  (25, 0.09) |
|  | post | 1.13  (26, 4.31) | 0.17  (33, 0.19) | 0.19  (27, 0.23) | 0.12  (25, 0.09) | 0.09  (27, 0.10) | 0.12  (31, 0.08) | 0.10  (31, 0.11) | 0.08  (24, 0.07) |
| Testosterone  (ng/ml) | pre | 0.27  (26, 0.15) | 0.26  (32, 0.11) | 0.23  (25, 0.15) | 0.26  (23, 0.14) | 4.21  (26, 1.35) | 5.33  (31, 1.85) | 4.64  (32, 1.45) | 4.44  (25, 1.77) |
|  | post | 0.23  (26, 0.14) | 0.24  (32, 0.10) | 0.21  (25, 0.15) | 0.23  (22, 0.11) | 4.58  (27, 1.67) | 5.50  (31, 1.98) | 4.39  (31, 1.73) | 4.50  (24, 1.61) |

*Notes.* pre; pretreatment; post, 4.5 hours after the gel administration; PLC_tra_ = transdermal placebo gel; PLC_int_ = intranasal placebo; OXT_int_ = intranasal oxytocin; EST_tra_ = transdermal estradiol.

**Table S2.** Oxytocin baseline and post treatment concentrations

|  | Females | | | | Males | | | |
| --- | --- | --- | --- | --- | --- | --- | --- | --- |
|  | PLC_tra_ & PLC_int_  Mean  (n, ± SD) | PLC_tra_ & OXT_int_  Mean  (n, ± SD) | EST_tra_ & PLC_int_  Mean  (n, ± SD) | EST_tra_ & OXT_int_  Mean  (n, ± SD) | PLC_tra_ & PLC_int_  Mean  (n, ± SD) | PLC_tra_ & OXT_int_  Mean  (n, ± SD) | EST_tra_ & PLC_int_  Mean  (n, ± SD) | EST_tra_ & OXT_int_  Mean  (n, ± SD) |
| Oxytocin pre  (pg/ml) | 2.03  (26, 0.72) | 1.51  (32, 0.59) | 1.65  (26, 0.65) | 1.63  (24, 0.52) | 1.90  (27, 0.79) | 2.02  (31, 0.83) | 1.96  (31, 0.68) | 1.97  (26, 0.67) |
| Oxytocin post  (pg/ml) | 2.14  (26, 0.69) | 4.92  (33, 1.71) | 1.83  (27, 0.62) | 4.84  (24, 1.74) | 2.09  (27, 0.89) | 6.03  (31, 2.71) | 2.25  (31, 0.61) | 6.28  (26, 3.18) |

*Notes.* pre; pretreatment; post, 4.5 hours after the gel administration; PLC_tra_ = transdermal placebo gel; PLC_int_ = intranasal placebo; OXT_int_ = intranasal oxytocin; EST_tra_ = transdermal estradiol.

**Table S3.** Demographic and psychometric baseline characteristics

|  | Females | | | | Males | | | |
| --- | --- | --- | --- | --- | --- | --- | --- | --- |
|  | PLC_tra_ & PLC_int_  Mean  (n, ± SD)x | PLC_tra_ & OXT_int_  Mean  (n, ± SD) | EST_tra_ & PLC_int_  Mean  (n, ± SD) | EST_tra_ & OXT_int_  Mean  (n, ± SD) | PLC_tra_ & PLC_int_  Mean  (n, ± SD) | PLC_tra_ & OXT_int_  Mean  (n, ± SD) | EST_tra_ & PLC_int_  Mean  (n, ± SD) | EST_tra_ & OXT_int_  Mean  (n, ± SD) |
| Age  (years) | 24.31  (26, 5.37) | 24.24  (33, 4.95) | 24.74  (27, 4.99) | 25.28  (25, 5.86) | 25.78  (27, 5.47) | 24.97  (31, 3.64) | 26.69  (32, 4.00) | 25.62  (26, 5.29) |
| Depressive symptoms (BDI^b^) | 4.00  (25, 3.44) | 1.84  (31, 2.33) | 3.20  (25, 3.59) | 3.40  (25, 3.24) | 3.46  (26, 5.62) | 1.83  (30, 2.61) | 2.00  (32, 2.31) | 2.63  (24, 2.90) |
| Autistic-like traits (AQ^c^) | 17.32  (25, 6.55) | 13.74  (31, 3.32) | 14.59  (27, 5.50) | 18.04  (25, 6.82) | 16.35  (26, 5.05) | 14.39  (31, 5.35) | 14.66  (32, 4.69) | 15.12  (26, 5.74) |
| Alexithymia (TAS^d^) | 45.80  (25, 9.79) | 42.26  (31, 10.69) | 43.67  (27, 12.58) | 44.4  (25, 10.21) | 41.23  (26, 9.09) | 43.52  (31, 9.32) | 41.81  (32, 8.23) | 39.77  (26, 9.58) |
| Trait anxiety (STAI^e^) | 38.52  (25, 8.27) | 35.48  (31, 7.65) | 33.89  (27, 7.58) | 38.08  (25, 9.70) | 33.00  (26, 8.39) | 33.06  (31, 6.62) | 33.94  (32, 6.70) | 32.16  (25, 6.82) |
| Social anxiety (Liebowitz Total^f^) | 20.88  (25, 10.54) | 18.39  (31, 11.97) | 17.41  (27, 11.93) | 19.80  (25, 11.30) | 13.12  (26, 13.09) | 11.81  (31, 10.56) | 12.09  (32, 10.46) | 13.58  (26, 13.32) |

*Notes.* Subjects rated their depressive symptoms with the ^b^ BDI (Becks Depression Inventory, Beck et al., 1996). Autistic-like traits were measured with the ^c^ AQ (Autism Spectrum Quotient, Baron-Cohen et al., 2006). Alexithymia was assessed with the ^d^ TAS (Toronto Alexithymia Scale, Taylor et al., 1985). The ^e^ STAI-Trait (State-Trait-Anxiety Inventory, Spielberger, 1970) was used to assess trait anxiety and the ^f^ Liebowitz questionnaire was used to measure social anxiety. PLC_tra_ = transdermal placebo gel; PLC_int_ = intranasal placebo; OXT_int_ = intranasal oxytocin; EST_tra_ = transdermal estradiol.

**Table S4.** Mood measurements

|  | Females | | | | Males | | | |
| --- | --- | --- | --- | --- | --- | --- | --- | --- |
|  | PLC_tra_ & PLC_int_  Mean  (n, ± SD) | PLC_tra_ & OXT_int_  Mean  (n, ± SD) | EST_tra_ & PLC_int_  Mean  (n, ± SD) | EST_tra_ & OXT_int_  Mean  (n, ± SD) | PLC_tra_ & PLC_int_  Mean  (n, ± SD) | PLC_tra_ & OXT_int_  Mean  (n, ± SD) | EST_tra_ & PLC_int_  Mean  (n, ± SD) | EST_tra_ & OXT_int_  Mean  (n, ± SD) |
| Positive affect pre | 27.28  (25, 6.69) | 27.44  (32, 6.61) | 29.26  (27, 5.54) | 27.84  (25, 5.37) | 30.00  (26, 5.25) | 29.52  (31, 5.99) | 28.44  (32, 5.92) | 31.12  (26, 6.30) |
| Positive affect post | 23.33  (24, 7.11) | 23.29  (31, 6.44) | 23.65  (26, 5.32) | 24.48  (25, 6.30) | 27.72  (25, 5.88) | 25.48  (29, 6.12) | 25.81  (32, 6.41) | 27.64  (25, 7.37) |
| Negative affect pre | 13.36  (25, 5.07) | 11.31  (32, 1.45) | 12.44  (27, 4.69) | 12.64  (25, 4.37) | 12.38  (26, 3.10) | 11.97  (31, 3.15) | 11.66  (32, 2.22) | 11.85  (26, 2.48) |
| Negative affect post | 12.42  (24, 2.15) | 11.23  (31, 1.76) | 11.62  (26, 2.19) | 12.28  (25, 2.61) | 11.16  (25, 1.57) | 11.34  (29, 2.16) | 11.19  (32, 1.77) | 11.40  (25, 3.03) |

*Notes.* Mood was assessed with the Positive and Negative Affect Schedule (PANAS). Abbreviations: pre; pretreatment; post, 4.5 hours after the gel administration; PLC_tra_ = transdermal placebo gel; PLC_int_ = intranasal placebo; OXT_int_ = intranasal oxytocin; EST_tra_ = transdermal estradiol.
